# Prediction of Chronic Obstructive Pulmonary Disease Using Machine Learning, Clinical Summary Notes, and Vital Signs: A Single-Center Retrospective Cohort Study in the United States

**DOI:** 10.3390/arm94010005

**Published:** 2026-01-07

**Authors:** Sabrina Meng, Hersh Sagreiya, Negar Orangi-Fard

**Affiliations:** 1 Perelman School of Medicine, University of Pennsylvania, Philadelphia, PA 19104, USA; 2 Department of Radiology, University of Pennsylvania, Philadelphia, PA 19104, USA; hersh.sagreiya@pennmedicine.upenn.edu; 3 Department of Mathematics and Statistics, Georgia Gwinnett College, Lawrenceville, GA 30043, USA

**Keywords:** chronic obstructive pulmonary disease, pulmonary medicine, artificial intelligence, machine learning, natural language processing, retrospective cohort study, United States

## Abstract

**Highlights:**

**What are the main findings?**

- Of the COPD exacerbation predictive models designed and assessed in our study, the clinical note-based support vector machine model achieved an AUC of 0.81 and accuracy of 84.0% in predicting COPD exacerbations.

**What are the implications of the main findings?**

- Clinically available patient data, clinical notes, and vital signs can effectively predict COPD exacerbations, potentially enabling earlier interventions, improved outcomes, and reduced healthcare burden.
- Integration of unstructured clinical notes with structured vital signs data using ML frameworks may improve early detection of COPD exacerbation risk.

**Abstract:**

**Introduction:** Chronic obstructive pulmonary disease (COPD) is a leading cause of morbidity and mortality. Early identification and timely intervention for COPD exacerbations can reduce hospitalizations and complications, as well as improve patient outcomes. **Methods:** To develop and evaluate predictive models for COPD exacerbations using machine learning (ML), we performed a retrospective study using intensive care unit patient records. Records including 31,667 clinical notes and 10,489 vital signs were used to train and validate two machine learning models to predict COPD exacerbations in patients with known or suspected COPD. Predictive performance was evaluated for support vector machine, quadratic discriminant analysis, and adaptive boosting algorithms using area under the receiver operating characteristic curve (AUC). **Results:** The clinical note-based support vector machine model achieved an AUC of 0.81 and accuracy of 84.0% in predicting COPD exacerbations. Data from patient monitors and hospital information systems provided sufficient information for accurate prediction, demonstrating the utility of combining physiological signals with clinical text data. **Discussion:** Clinically available patient data and vital signs can effectively predict COPD exacerbations, potentially enabling earlier interventions, improved outcomes, and reduced healthcare burden. These findings suggest that integrating unstructured clinical notes with structured vital signs using ML frameworks may improve early detection of exacerbation risk, thus enabling appropriate patient counseling, triage, and treatment based on COPD severity.

## 1. Introduction

Chronic obstructive pulmonary disease (COPD) is a highly prevalent disease characterized by persistent respiratory symptoms and airflow limitations. Specifically, COPD is defined as a reduction in FEV1/FVC, where FEV1 represents forced expiratory volume in one second, and FVC represents forced vital capacity [1]. This ratio reflects the amount of air that can be forcefully exhaled from the lungs and is measured using spirometry.

### 1.1. Epidemiology and Risk Factors

As of 2020, COPD has become the sixth leading cause of death in the United States and results in most deaths related to chronic lower respiratory diseases [2]. The prevalence of COPD in the United States has remained stable at approximately 6% from 2011 to 2021 [3]. One study using data from across 46 US states and New York City found that approximately 10.3% of death certificates list COPD as the cause of death [2]. The global burden of COPD is high, and in 2019, the World Health Organization estimated COPD to be the third leading cause of death worldwide [4]. In 2021, 213.39 million prevalent cases of COPD were estimated, for a prevalence rate of 2.5%, and it is estimated that 3.72 million deaths were due to COPD [5]. Additionally, COPD places a large burden on the healthcare system because hospitalization in individuals with advanced COPD is common and recurrent, with 90-day readmission rates ranging from 16–48% [6]. The length of each hospital admission is also elevated, with a mean length of 9 days [6]. Multiple risk factors for COPD have been identified, including male sex, smoking, advanced age, body mass index of less than 18.5 kg/m^2^, childhood hospital admission for severe respiratory disease, childhood asthma, family history of obstructive lung disease, history of tuberculosis, biomass exposure, occupational exposure to dust or smoke, and ambient air pollution [7,8].

### 1.2. Pathophysiology

The pathophysiology of COPD is complex and involves multiple factors, including elastic fiber breakdown in alveoli, diaphragmatic dysfunction, pulmonary arterial hypertension, reactive oxygen species, and inflammatory processes [9]. In normal lungs, elastic fibers are present to allow for passive recoil; however, emphysema, a main phenotype of COPD, is characterized by degeneration of these elastic fibers resulting in airflow limitation [10,11]. Limited airflow also leads to trapping of air in the lungs, resulting in hyperinflation, mechanical disadvantage of the diaphragm, and respiratory dysfunction [12]. Long-term respiratory dysfunction may also contribute to vascular remodeling and pulmonary arterial hypertension, which is a major contributor to COPD exacerbations [13]. A variety of inflammatory processes are implicated in COPD pathogenesis, including macrophages, CD8^+^ T-cells, neutrophils, and alveolar epithelial cells, which release a variety of inflammatory mediators, such as perforins, TNF-α, leukotriene B4, IL-8, and others [9]. Crucial to the pathogenesis of COPD is an imbalance of proteases that degrade elastin, such as neutrophil elastase, and protease inhibitors, such as α1-antitrypsin [14,15]. This leads to symptoms of respiratory decline as described above, as well as oxidative stress that causes further tissue damage [16]. Another major contributor of oxidative damage in COPD is cigarette smoke [16].

### 1.3. Diagnosis, Management, and Multidisciplinary Care

COPD is diagnosed when the FEV1/FVC ratio measured by spirometry is irreversible after the administration of a bronchodilator and is less than the lower limit of normal as defined by the Global Lung Function Initiative reference values [17,18]. However, if normal values are not available, then a fixed standard of FEV1/FVC < 0.7 can be used, and other techniques such as blood gas analysis, whole body plethysmography, and chest radiographs can be performed for further characterization of COPD [17]. According to the Global Initiative for Chronic Obstructive Lung Disease (GOLD) guidelines, COPD may be classified based on etiology, including genetic COPD, COPD resulting from abnormal lung development, environmental COPD, COPD due to infections, COPD associated with asthma, and COPD of unknown cause [19]. COPD may also be graded based on both severity (GOLD grades 1–4) and exacerbation history (GOLD groups A–D), with the latter being an important tool for guiding treatment [19]. Comorbidities of COPD span both pulmonary and extrapulmonary conditions, including asthma, bronchiectasis, lung cancer, cardiovascular disease, renal dysfunction, psychiatric disorders, obstructive sleep apnea, osteoporosis, and diabetes mellitus [17].

Treatment of COPD is variable and dictated by the severity of disease. For patients classified as group A, a bronchodilator is prescribed as initial therapy, with possible follow-up treatment with a long-acting ß-agonist (LABA) or long-acting muscarinic antagonist (LAMA). Those classified under Group B receive a LABA, LAMA, or LABA/LAMA as initial therapy, with LABA/LAMA as follow-up therapy. Finally, those classified as Group E (formerly C and D) typically receive LABA/LAMA/inhaled corticosteroids (ICS) as initial therapy, with additional medications such as roflumilast and azithromycin added for follow-up therapy [17,19]. Because COPD patients often face increased energy demands, systemic inflammation, and reduced dietary intake, nutritional screening measures and supplemental nutrition such as amino acids, omega-3 fatty acids, vitamin D, and antioxidants may be warranted [20,21]. Strategies can also be implemented to prevent the development and progression of COPD, which involves actions such as controlling cigarette smoking, reducing environmental pollution, early diagnosis, medication to reduce disease progression, multidisciplinary care to reduce the extrapulmonary burdens of COPD, and preventing exacerbations, which have been identified as important drivers of disease progression [22].

A COPD exacerbation, or flare-up, occurs when COPD respiratory symptoms acutely become more severe compared to baseline. Several definitions of COPD exacerbations have been described, including increased shortness of breath, increased sputum production, respiratory deterioration that requires changes to normal treatment, and chest illness that causes patients to lose time from work or to go to bed [23]. A considerable percentage of COPD patients, approximately 22% of those with moderate COPD, experience periodic exacerbations of symptoms, which are serious threats to patients and may increase mortality risk [24,25]. Exacerbations reflect accelerated declines in lung function, which can result in permanent functional decline in patients and are associated with an increased risk of rehospitalization and death [24,26].

Due to the high burden of COPD and its associated exacerbations, there is great interest in preventing their occurrence. In clinical practice, “frequent exacerbators” are defined as individuals who have at least two moderate COPD exacerbations or one severe COPD exacerbation per year [27]. The number of exacerbations and status as a “frequent exacerbator” is used to guide therapeutic choices for exacerbation prevention, such as the administration of inhaled corticosteroids (ICS) [27,28].

While there exist guidelines regarding the clinical criteria (e.g., dyspnea, heart rate, respiratory rate, etc.) necessary to define different types of exacerbations, there are limitations to this approach [19]. Many variables can contribute to COPD exacerbations, and the data from a small subset of clinically measured values may not accurately characterize the exacerbation. For example, the impact of other contexts of a patient’s condition, such as demographic variables or comorbid conditions, may be difficult to assess using clinical guidelines.

### 1.4. Current COPD Prediction Models

Several predictive machine learning (ML) models have been created to leverage computational power and large clinical databases to improve the approach for predicting and characterizing COPD exacerbations. One example is the Acute COPD Exacerbation Prediction Tool (ACCEPT), a cohort study that used a mixed-effects model to predict COPD exacerbations over one year; however, this study is limited by the availability of variables such as comorbid conditions [29]. Moreover, Matheson et al. (2018) reviewed and assessed published models for predicting COPD development. They identified 4481 records and selected 30 articles for full-text review; however, only four models aimed to predict an individual’s future risk of COPD [30]. Another review study by Guerra et al. (2017) examined 1382 studies, of which 25 studies with 27 prediction models were selected for evaluation of their performance in predicting COPD exacerbation [31]. However, these models were limited by the availability of predictors, lack of external validation, and applicability to clinical care [31].

More recently, Moraza et al. (2025) used telemonitoring physiological data—breathing rate, heart rate, and oxygen saturation (SpO_2_)—combined with questionnaire responses in a home-based system to predict symptom deterioration within three days, achieving an AUC of 0.91 and an Area Under the Precision–Recall Curve (AUPRC) of 0.53 [32]. Similarly, Zhu et al. (2025) applied gradient boosting models to laboratory inflammatory biomarkers (e.g., neutrophil-to-lymphocyte ratio, monocyte-to-lymphocyte ratio, eosinophil-to-lymphocyte ratio) and demographic data, reaching an AUC of 0.90 and accuracy near 0.95 [33]. Atzeni et al. (2025) incorporated environmental exposure data from personal air quality monitors and daily symptom logs into random forest and XGBoost models, with AUCs up to 0.90 [34]. While these models demonstrate excellent performance, they also utilize predictors that are not available for all patients, such as data from inflammatory biomarkers, continuous environmental monitoring, and home-based care systems. Finally, Zhang et al. (2023) reviewed ML applications across spirometry, vital signs, imaging, biomarkers, and free-text notes, noting the potential of integrating multi-modal data for COPD prediction [35].

### 1.5. Research Aims

We propose a novel COPD Prediction Using ML (CPML) framework that predicts acute COPD exacerbations rather than COPD diagnoses using data commonly available for patients. This framework integrates both structured physiological signals and unstructured clinical notes to forecast flare-ups in ICU patients with known or suspected COPD. By utilizing a diverse set of physiological inputs and harnessing the rich information contained within free-text clinical notes, we hypothesize that our model will achieve robust performance in predicting COPD exacerbation. The primary objective of this study was to assess the performance of the CPML framework in predicting COPD exacerbations using both prediction accuracy and AUC values. Secondary objectives of the study included (1) exploring the feasibility of utilizing free text input in a COPD prediction model and (2) comparing the predictive performance of models utilizing free text input with conventional models utilizing structured data.

## 2. Materials and Methods

We used the MIMIC-III Clinical Database, a large, freely available database containing de-identified health-related data associated with over 40,000 patients who stayed in critical care units at Beth Israel Deaconess Medical Center in Boston, Massachusetts between 2001 and 2012 [36,37]. In our study, we use the ADMISSIONS table, which consists of 58,976 entries containing admission information, and the NOTEEVENTS table, which has 2,083,180 entries containing clinical free text notes for each hospitalization, from the MIMIC-III Clinical Database [37,38]. We additionally used the MIMIC-III Waveform Database Matched Subset containing 22,317 waveform records and 22,247 numeric records from 10,282 distinct ICU patients. These records include digitized signals such as electrocardiogram (ECG), arterial blood pressure (ABP), respiratory rate and photoplethysmography (PPG), as well as periodic measurements of vital signs including heart rate, SpO_2_, and systolic, mean, and diastolic blood pressure.

Patients with known or suspected COPD were identified using COPD-related ICD diagnostic codes supplemented by supporting clinical documentation within ICU notes. Baseline COPD diagnosis was distinguished from acute COPD exacerbation, which was defined as an episode of acute respiratory worsening meeting the outcome criteria described above. Episodes of general respiratory deterioration without evidence specific to COPD exacerbation were not labeled as outcome events.

Due to data use restrictions associated with the MIMIC-III database, patient-level data and executable code cannot be publicly shared. However, the full modeling pipeline—including data preprocessing, feature extraction, model training, and evaluation procedures—is described in sufficient detail in below to enable methodological replication.

**Figure 1:**
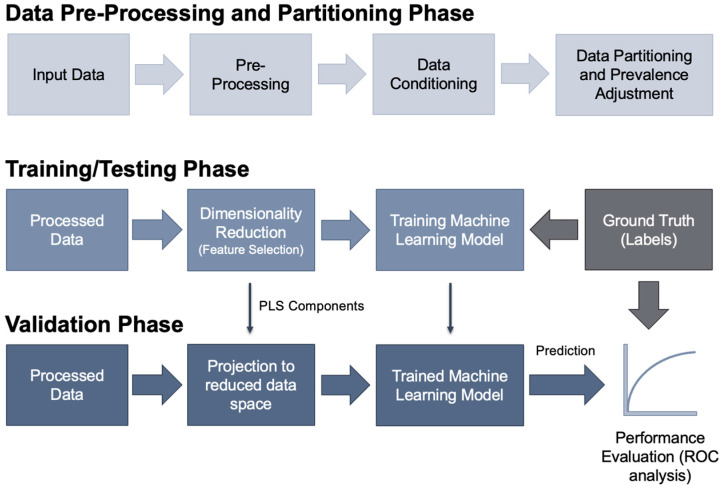
CPML framework for predicting flare-ups in COPD patients. First, data is pre-processed to extract the relevant information, partition the entries, and define the appropriate variables. Next, the processed data enters the training and testing phase, where dimensionality reduction is completed to encode key features in a machine-understandable format, and the reduced data is passed to the machine learning model for training. During this process, ground truth labels are also supplied to the model, which enables model learning. Finally, processed and reduced data is given as input to the trained model, which outputs a prediction that is compared to a ground truth label, enabling evaluation of its performance via strategies such as receiver operating curve (ROC) analysis.

CPML is a systematic framework that uses both structured data, such as vital signs and lab results, and unstructured data, such as clinical notes, to extract relevant features that predict COPD exacerbations. The framework includes data pre-processing, feature engineering guided by clinical guidelines (e.g., GOLD staging), and model training and validation to create robust predictive models that can anticipate patient flare-ups in real time. Two predictive models following the CPML framework were developed: one utilizing features derived from clinical notes from the MIMIC-III Clinical Database as input and another utilizing features derived from vital signs from the MIMIC-III Waveform Database Matched Subset as input. Both models were designed to predict the risk of acute COPD exacerbations in patients with known or suspected COPD, not to diagnose COPD itself. We emphasize that predictor variables were temporally aligned using a fixed prediction horizon preceding the onset of COPD exacerbation. Clinical notes and vital sign measurements were extracted from a predefined time window prior to the documented exacerbation event, ensuring that all predictors reflected information available before outcome occurrence and supporting real-time risk prediction. Prevalence adjustment and feature selection via partial least-squares (PLS) regression were applied to maximize prediction of exacerbation events, retaining features most associated with impending flare-ups. Figure 1 shows a block diagram of the CPML framework for predicting COPD flare-ups in patients.

**Figure 2:**
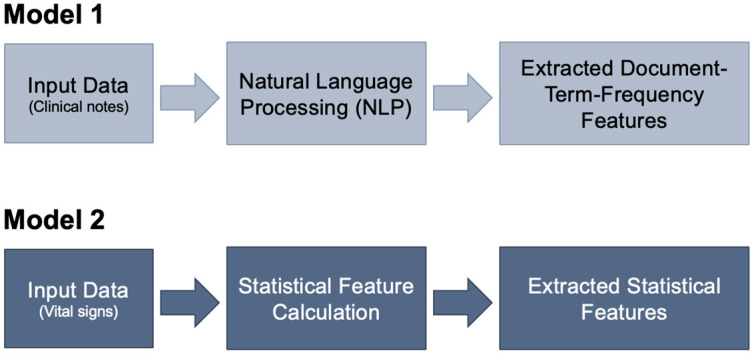
Data pre-processing steps for Model 1 (clinical notes) and Model 2 (vital signs) prior to input into the machine learning model. In Model 1, input data in the form of clinical notes is passed to a natural language processing (NLP) algorithm. This algorithm then extracts document-term-frequency features, which are numerical representations quantifying the occurrence and importance of terms within the note. In Model 2, input data in the form of vital signs undergoes processing to calculate statistical features, which are extracted.

Model 1 is based on free text clinical notes as input and first uses natural language processing (NLP) to convert the notes into numerical data (features) via bag-of-words tokenization and vectorization techniques before feeding the vectorized notes to a ML model for COPD exacerbation prediction [39]. Model 2 is based on vital signs as input, including heart rate, SpO_2_, and respiratory rate signals, as well as their derived statistical features: maximum, minimum, mean, median, and standard deviation. Additional features were extracted by categorizing the signals based on threshold values defined in GOLD staging definition [40]. The groupings for heart rate, measured in beats per minute, were “Normal” (<90), “Mild” (90–100), “Moderate” (100–110), “Severe” (110–120) and “Very Severe” (>120). For respiratory rate, measured in breaths per minute, the groupings are “Normal” (12–18), “Low” (<12), “High” (18–20), and “Abnormal” (>20). For SpO_2_, the groupings were “Normal” (>92%), “Mild” (90–92%), “Moderate” (85–90%), “Severe” (80–85%), and “Very Severe” (<80%). In summary, continuous vital sign variables were categorized using clinically meaningful thresholds informed by Global Initiative for Chronic Obstructive Lung Disease (GOLD) recommendations, where applicable. These thresholds were used to discretize variables into clinically interpretable ranges associated with disease severity and exacerbation risk. The two ML models and their corresponding data pre-processing techniques are summarized in Figure 2.

We compared the performance of three ML techniques for COPD prediction using each model: support vector machine (SVM), adaptive boosting (AdaBoost), and quadratic discriminant analysis (QDA) [41,42,43]. SVM projects the input features into a higher-dimensional space, where it finds an optimal hyperplane that maximizes the margin between the hyperplane and the closest data points [41]. In this study, we used a Gaussian radial basis function (RBF) kernel to extend the SVM for non-linear classification. AdaBoost is an ensemble technique that combines multiple weak classifiers, or decision trees, that work in conjunction to reach a final classification [42]. Finally, QDA uses a transformation function to maximize the ratio of between-class variance to within-class variance and to minimize the overlap of the transformed distributions [43]. In this study, a “pseudo-quadratic” (SQ) transformation was used. SQ uses an inverse covariance matrix as a cost function to measure the variability of covariance matrices among the classes. To evaluate the performance of each predictive model and ML technique combination, we calculated the Receiver Operating Characteristic (ROC) Area Under the Curve (AUC), which represents the performance of a binary classifier as its discrimination threshold is varied [44]. Prediction accuracies were calculated for each model and ML technique.

This observational study is reported in accordance with the Strengthening the Reporting of Observational Studies in Epidemiology (STROBE) guidelines.

## 3. Results

**Figure 3:**
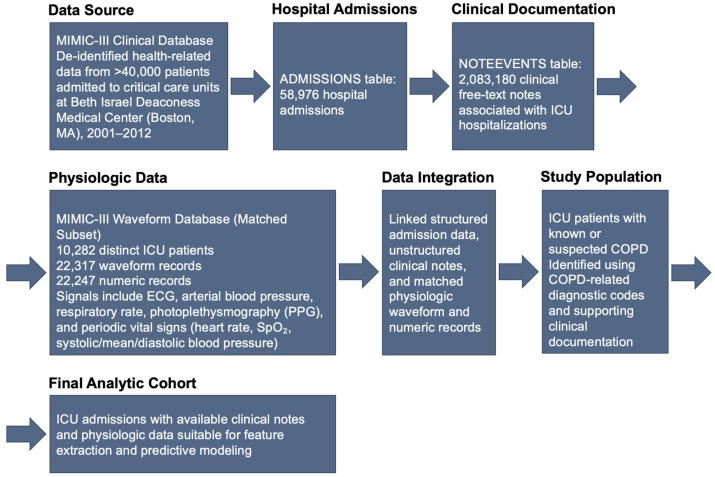
Study flow diagram illustrating cohort identification, inclusion and exclusion criteria, and the final analytic sample used in this study.

Figure 3 presents the study flow diagram illustrating cohort identification, inclusion and exclusion criteria, and the final analytic sample used in this study.

**Figure 4:**
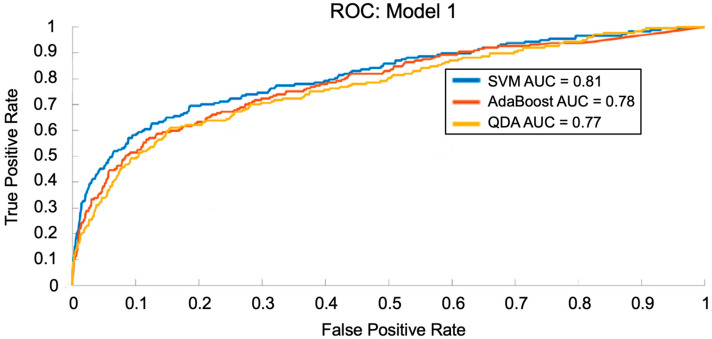
Receiver operating characteristic (ROC) curves for the SVM, AdaBoost, and QDA techniques with Model 1 using clinical note data to predict COPD exacerbations. The area under the curve (AUC), which measures how well a distinguishes between two groups (1 = perfect, 0.5 = random), was calculated for each machine learning technique, with SVM resulting in the greatest performance (0.81), followed by AdaBoost (0.78) and QDA (0.77).

To train and test Model 1, 31,667 records containing clinical notes from the MIMIC-III Clinical Database were used; these records represented 354 patients with COPD, with several records per patient [36,37]. 15,833 (50%) records were used for training, and 15,834 (50%) records were used for testing [36,37]. During the data pre-processing step aided by NLP, 3000 features were extracted, of which 15 were retained after dimensionality reduction by the PLS module. These 15 features were passed to the predictive model using the SVM, AdaBoost, and QDA techniques and evaluated for performance on COPD prediction. Figure 4 shows the ROCs for Model 1 using each ML technique. We found that the optimal performance was achieved using 15 PLS features. Table 1 shows the corresponding AUC and accuracy values on the test set for each ML technique using Model 1. SVM, AdaBoost, and QDA achieved AUC values of 0.81, 0.78, and 0.77, respectively, and accuracies of 84.0%, 78.2%, and 75.0%, respectively.

**Figure 5:**
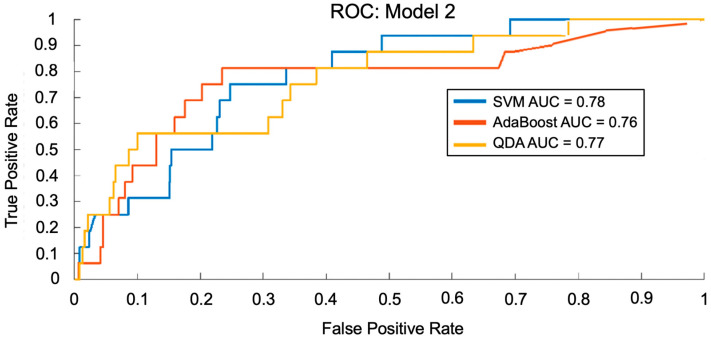
Receiver operating characteristic (ROC) curves for SVM, AdaBoost, and QDA techniques with Model 2 using vital signs to predict COPD exacerbations. The area under the curve (AUC), which measures how well the model distinguishes between two groups (1 = perfect, 0.5 = random), was calculated for each machine learning technique, with SVM resulting in the greatest performance (0.78), followed by QDA (0.77) and AdaBoost (0.76).

To train and test Model 2, 10,489 records containing vital signs data from the MIMIC-III Waveform Database Matched Subset were used, with 2551 records representing patients with COPD [37,38]. 5591 (70%) records were used for training, and 7938 (30%) records were used for testing [37,38]. After data pre-processing followed by dimensionality reduction by the PLS module, 15 features were passed to the predictive model using the SVM, AdaBoost, and QDA techniques for COPD prediction. Figure 5 shows the ROCs for Model 2 using each ML technique. Again, we found that the optimal performance was achieved using 15 PLS features. Table 2 shows the corresponding AUC and accuracy values on the test set for each ML technique using Model 2. SVM, AdaBoost, and QDA achieved AUCs of 0.78, 0.76, and 0.77, respectively, and accuracies of 77.0%, 83.0%, and 67.0%, respectively.

## 4. Discussion

COPD is a common disease that leads to declining respiratory function and airflow limitations. It is a significant cause of morbidity and mortality both in the United States and globally, and frequent hospital admissions contribute to the large healthcare burden [2,4,6]. COPD exacerbations, characterized by an acute decline in respiratory function from baseline, are debilitating and increase the risk of hospitalization or death [24,26]. As a result, predicting COPD exacerbations to better manage high-risk patients is paramount. While some clinical guidelines and predictive models exist, many present significant limitations.

In this study, we use respiratory clinical notes (Model 1) and vital signs (Model 2), correspondingly from the MIMIC-III Dataset and the MIMIC-III Waveform Database Matched Subset, to predict COPD exacerbations. For both types of input data, we compared the performance of three machine learning techniques: SVM, AdaBoost, and QDA [41,42,43]. After selecting the most important input features using PLS-based dimensionality reduction, Model 1 achieved a higher performance, attaining an AUC of 0.81, 0.78, and 0.77, and accuracy of 84.0%, 78.2%, and 75.0% using SVM, AdaBoost, and QDA, respectively. Model 2 achieved an AUC of 0.78, 0.76, and 0.77 and accuracy of 77.0%, 83.0%, and 67.0% using SVM, AdaBoost, and QDA, respectively. These results indicate that both structured and unstructured patient data can be leveraged to predict COPD exacerbations in real time, highlighting the value of integrating clinical notes and vital signs within the CPML framework.

### 4.1. Comparison with Current Literature

These results demonstrate competitive AUC and accuracy in COPD exacerbation prediction using the CPML framework compared to available models. For example, most currently published models report AUC values of 0.58–0.85 [29,30,31,45]. A recently published COPD prediction model achieved an AUC of up to 0.90 during testing [34]. However, the study utilized data collected from custom-made air sensors and daily COPD-status-related logs recorded by participants, such as Peak Expiratory Flow rates and sleep quality, which are not data available for most patients [34]. Thus, our research illustrates the promise of the CPML framework for COPD prediction using data commonly available in the health record. Moreover, while most existing models use structured data as inputs, our findings demonstrated improved model performance when using NLP to extract data from free-text clinical notes. Our results show the potential for improving the performance of existing models for COPD prediction by incorporating unstructured inputs and constructing multimodal predictive algorithms. We have additionally described and validated an NLP-assisted framework for accommodating unstructured inputs in predictive ML models.

Additionally, our findings can be integrated with existing COPD prediction frameworks to further improve performance and utility of our model. Comparable short-term prediction work by Moraza et al. (2025) using telemonitoring vital signs (breathing rate, heart rate, SpO_2_) and home questionnaire data, achieved an AUC of 0.91, highlighting the predictive value of physiologic monitoring outside of hospital settings [32]. Zhu et al. (2025) demonstrated high accuracy in COPD exacerbation prediction using laboratory inflammatory biomarkers, showing a complementary path for models built on routinely collected lab data [33]. Atzeni et al. (2025) leveraged environmental exposure data and daily symptom logs in a wearable-sensor framework, reaching an AUC of 0.90—indicating the potential for integrating environmental monitoring with physiologic data [34]. Finally, the review by Zhang et al. (2023) underscores the promise of multi-modal approaches combining structured, unstructured, physiologic, and environmental inputs—supporting the rationale for our CPML framework’s dual-input design [35]. Recently, developments in computer vision and deep learning have enabled advanced image analysis, enabling the extraction of vascular biomarkers from routine computed tomography (CT) scans [46,47]. These advances allow clinicians to make use of subtle biomarkers invisible to the human eye, enabling richer disease stratification frameworks, improved prognostic capabilities, and individualized treatment [48,49]. In the future, the CPML framework can be augmented with these predictors, further improving predictive performance for patients who have available data for these predictors.

To our knowledge, our model is one of the first to include free-text clinical notes as an input to a COPD exacerbation prediction model. Integration of free text and structured data has been done in other clinical prediction models with great success. For example, one study reviewed 126 studies describing 145 clinical prediction problems and found that the use of unstructured text in addition to structured data was beneficial for clinical prediction models in most cases [50]. Another study evaluating the usage of artificial intelligence in the prediction of sepsis found that mining unstructured clinical notes improved the algorithm’s accuracy when compared to using only clinical measurements as input [51]. Yet another study investigating the role of ML in predicting medical emergency severity in emergency department patients integrated structured and unstructured data (e.g., chief complaints and reasons for visit) processed by transformer-based NLP models, which similarly demonstrated that the combination of structured and unstructured data types improved the prediction capabilities of the model [52]. From these studies, it can be shown that the addition of unstructured data as an input often improves the performance of clinical predictive models. Thus, our clinical notes-based model represents a significant advance in predictive ML models for COPD exacerbation prediction.

### 4.2. Opportunities for Future Work

In the future, other sources of input can be introduced into the model to improve performance, including laboratory values, environmental conditions, imaging reports and data, progress notes, ventilator settings, etc. This is especially promising since previous studies have demonstrated improved accuracy of clinical predictive models when structured and unstructured data are combined. Additionally, it may be valuable to explore the potential of the CPML framework in predicting other COPD-related metrics, such as length of stay, readmission risk, and survival.

Recently, there have also been notable developments in ML and NLP frameworks, which present an exciting opportunity for embedding into our CPML framework. For example, while we used classic ML approaches in our model (SVM, AdaBoost, and QDA), there are newer techniques based upon neural networks, deep learning, representation learning, and other multimodal approaches, which offer the opportunity for enhanced performance. In a study comparing the performance of classical ML and deep learning techniques for predicting lung cancer survival, it was found that deep learning surpassed the performance of multiple traditional ML models [53]. Similar results have also been found in disciplines outside of respiratory medicine: When researchers compared conventional learning and deep learning algorithms for Crohn’s disease, they found that a deep learning algorithm using a recurrent neural network achieved the highest AUC out of all assessed models [54].

Moreover, in addition to the bag-of-words approach to NLP used in our study, methodologies based on word embeddings and contextual embeddings have grown in popularity in recent years and have demonstrated excellent performance. One class of language embedding techniques is word embedding (e.g., word2vec and Global Vectors for Word Representation [GloVe]), which is motivated by deep learning and has shown promise over the bag-of-words approach [55,56]. For example, studies have found that word2vec can outperform bag-of-words on certain clinical text classification tasks, although the performance of such embeddings may vary depending on the source of training (e.g., electronic health record, medical literature, and pre-trained word embeddings) [55,56]. Another class of language embedding techniques is contextual embedding, which uses transformer-based architectures to enable context awareness and includes models such as Bidirectional Encoder Representations from Transformers (BERT), Pathways Language Model (PaLM), and Generative Pre-trained Transformer (GPT). These methods present an advantage over word embedding techniques, with BERT outperforming word2vec on a task involving extraction and validation of semantic features from a transcribed clinical encounter [57]. Other studies compared the performance of BERT, PaLM, and GPT in identifying medication names, routes, and frequencies in ophthalmology progress notes and found that PaLM and GPT offer improved performance over BERT [58]. Of the BERT models, BioBERT achieved the highest performance, and GPT-4 achieved the best performance out of all assessed models [58]. Thus, there is an opportunity to further improve the performance of the CPML framework reported in our study given recent advances in ML and NLP technologies.

### 4.3. Perspective for Clinical and Assistive Practice

Our COPD prediction model has the potential to impact care for COPD patients across many specialties and care contexts. For example, it has been suggested that patients who frequently have exacerbations should be initiated on ICS, whereas treatment pathways targeting only dyspnea do not typically emphasize ICS [27,28]. Because frequent exacerbations are associated with greater COPD severity and increased risk of progression, our model has the potential to detect exacerbation risk early, thus enabling treatment to reduce the number of future exacerbations and other supportive management techniques like nutritional supplementation [20,21,22]. Patients who experience frequent exacerbations may also benefit from pulmonary rehabilitation therapy [28]. As a result, COPD exacerbation risk prediction can help pulmonologists and other multidisciplinary providers make informed treatment plans for patients. Moreover, when patients present with a COPD exacerbation, only a fraction of them are admitted for treatment, with a study based in the United States finding that 51% of patients were hospitalized in 2010 and 31% in 2018 [59]. However, patients with a high risk for exacerbations are typically associated with higher rates of hospitalization compared to those at low risk for exacerbations [60]. Thus, the ability to identify patients at high risk of exacerbation may help with triage of patients presenting for emergency care and the decision to admit them for inpatient care. Risk stratification can also be useful for patient counseling, such as the creation of individualized action plans for COPD exacerbations and referral to other specialty providers to monitor for extrapulmonary comorbidities of COPD.

### 4.4. Limitations and Strengths

Study limitations include a lack of external validation on patients outside of the MIMIC-III dataset, which consists of patients from a single medical center in the United States, limiting the generalizability of our findings. Future studies can utilize data across multiple centers consisting of patient populations both in the United States and abroad to improve generalizability. Different study designs, such as prospective cohort studies, may also be considered for external validity and to better characterize the impact of the CPML model on patient management and outcomes. Another limitation of our study was the small subset of ML approaches used: SVM, AdaBoost, and QDA. Future work is warranted to explore the performance of the CPML model in conjunction with other ML techniques, including those based on neural networks, deep learning, representation learning, and multimodal approaches. Moreover, the impact of recent NLP methodologies, such as novel embedding techniques and large language models (e.g., BioBERT, Med-PaLM, and GPT-4), on the predictive performance of our model should be assessed [61,62,63].

Our study is strengthened by a large sample size of patients and longitudinal data collection. Additionally, parameters used as inputs to our models—clinical notes and vital signs—are commonly collected and easily accessible within the health record; our usage of unstructured notes not only improves model performance but also minimizes the data processing needed to extract relevant information. Collectively, these findings emphasize that the CPML framework has the potential to quickly and accurately identify those at risk for imminent COPD exacerbations, allowing earlier clinical interventions.

## 5. Conclusions

In conclusion, COPD is a common disorder that leads to high morbidity and mortality, especially in those who experience frequent exacerbations. While some clinical guidelines and predictive models have been developed to better characterize and predict COPD exacerbations, these approaches have limitations. In this study, we design and validate a CPML framework that utilizes (i) respiratory clinical notes and (ii) vital signs as input. Our CPML framework demonstrated high AUC and accuracy for predicting COPD exacerbation, and the combination of clinical notes as the input and SVM as the machine learning technique achieved the highest performance. These findings suggest that our NLP- and vitals-assisted CPML framework may improve early detection of COPD exacerbation risk, thus enabling appropriate patient counseling, triage, and treatment based on COPD severity. Further studies using more varied populations are warranted to improve the generalizability of our findings and prepare the technology for broad clinical utility.

## Figures and Tables

**Table 1:** Accuracy and AUC of each evaluated machine learning technique for Model 1.

Technique	Accuracy	AUC
SVM	84.0%	0.81
AdaBoost	78.2%	0.78
QDA	75.0%	0.77

**Table 2:** Accuracy and AUC of each evaluated machine learning technique for Model 2.

Technique	Accuracy	AUC
SVM	77.0%	0.78
AdaBoost	83.0%	0.76
QDA	67.0%	0.77

## Data Availability

The original data presented in the study are openly available in the MIMIC-III Clinical Database, a large database containing de-identified health-related data associated with over 40,000 patients, at https://doi.org/10.13026/C2XW26.

## Abbreviations

The following abbreviations are used in this manuscript:

COPD	Chronic obstructive pulmonary disease
GOLD	Global Initiative for Chronic Obstructive Lung Disease
LABA	Long-acting ß-agonist
LAMA	Long-acting muscarinic antagonist
ICS	Inhaled corticosteroids
NLP	Natural language processing
ICU	Intensive care unit
AUC	Area under the receiver operating characteristic curve
SVM	Support vector machine
QDA	Quadratic discriminant analysis
AdaBoost	Adaptive boosting
FEV1	Forced expiratory volume in one second
FVC	Forced vital capacity
ACCEPT	Acute COPD Exacerbation Prediction Tool
SpO_2_	Oxygen saturation
AUPRC	Area under the precision–recall curve
XGBoost	Extreme Gradient Boosting
CPML	COPD Prediction Using ML
ECG	Electrocardiogram
ABP	Arterial blood pressure
PPG	Photoplethysmography
PLS	Partial least-squares
ROC	Receiver Operating Characteristic
CT	Computed Tomography
GloVe	Global Vectors for Word Representation
BERT	Bidirectional Encoder Representations from Transformers
PaLM	Pathways Language Model
GPT	Generative Pre-trained Transformer
